# Equine tapeworm (*Anoplocephala* spp.) infection: evaluation of saliva- and serum-based antibody detection methods and risk factor analysis in Slovak horse populations

**DOI:** 10.1007/s00436-023-07994-1

**Published:** 2023-10-07

**Authors:** Ludmila Burcáková, Alzbeta Königová, Tetiana A. Kuzmina, Corrine J. Austin, Jacqueline B. Matthews, Kirsty L. Lightbody, Natalia A. Peczak, Yaroslav Syrota, Marian Várady

**Affiliations:** 1 Institute of Parasitology, Slovak Academy of Sciences, Hlinkova 3, Kosice, 04001 Slovakia; 2 University of Veterinary Medicine and Pharmacy in Kosice, Komenskeho 73, Kosice, 04181 Slovakia; 3 I. I. Schmalhausen Institute of Zoology NAS of Ukraine, Bogdan Khmelnytsky Street, 15, Kyiv, 01054 Ukraine; 4 Austin Davis Biologics Ltd, Unit 1 Denfield Lodge, Lower Street, Great Addington, Northants, NN14 4BL UK; 5 African Amphibian Conservation Research Group, Unit for Environmental Sciences and Management, North-West University, Potchefstroom, South Africa

**Keywords:** Horses, *Anoplocephala*, Coprological methods, ELISA, Tapeworm infection, Risk factor analysis

## Abstract

A lack of accurate information on the prevalence and distribution of *Anoplocephala* spp. infections on horse farms has led to insufficient attention to tapeworm control and increasing horse anoplocephaloses in Europe. Our study aimed to examine the occurrence of *Anoplocephala* spp. infection using coprological, serum- and saliva-based antibody detection methods and to analyze the risk factors associated with tapeworm infection in domestic horses in Slovakia. Fecal, serum, and saliva samples were collected from 427 horses from 31 farms in Slovakia. Additionally, a questionnaire study was conducted to collect information on tapeworm distribution on horse farms and analyze risk factors associated with infection. Fecal samples were examined by the mini-FLOTAC and the double centrifugation/combined sedimentation-flotation techniques. Serum and saliva samples were analyzed by ELISA to determine antibody levels against *Anoplocephala* spp. The effects of variables associated with an individual horse were tested for the positive result of the saliva ELISA test on *Anoplocephala* spp. Cestode eggs were detected in 1.99% of fecal samples (farm prevalence 12.90%), with no differences between the two coprological methods. Serum-based tapeworm ELISA results revealed that 39.39% of horses tested positive (farm prevalence 83.87%); while saliva-based tapeworm ELISA results revealed 56.95% positive horses (farm prevalence 96.77%). Binary logistic regression analysis revealed four meaningful predictors that significantly impacted the likelihood of detecting tapeworm infection in horses: horse age, pasture size, anthelmintic treatment scheme, and access to pasture. The influences of other variables associated with an individual horse were not significantly associated with detecting tapeworm infection.

## Introduction

Horses with access to pasture that graze year-round or daily at specific intervals are exposed to a range of gastrointestinal helminths (Stratford et al. 2014). In the past, most attention was given to strongyles and *Parascaris* spp., nematodes considered the main pathogenic parasitic agents affecting equids (Drudge and Lyons 1977; Lyons et al. 1992; Nielsen et al. 2016). The high prevalence and potential pathogenicity of strongylid nematodes led to a focus on, and widespread use of anthelmintics (in particular, macrocyclic lactones) directed against these types of helminths (Nielsen et al. 2016). Historically, less attention was paid to equine tapeworms, until the emergence of reports that described the clinical impact of these parasites in horses that grazed heavily contaminated pastures, with effects including spasmodic colic, and intestinal and peritoneal impactions with potentially fatal consequences (Owen et al. 1989; Proudman and Edwards 1992; Proudman et al. 1998). Such effects were attributed to high tapeworm burdens (Pearson et al. 1993; Gasser et al. 2005; Veronesi et al. 2009; Pavone et al. 2011; Back et al. 2013; Pittaway et al. 2014). The most pathogenic and abundant tapeworm species in horses is *Anoplocephala perfoliata* (Goeze, 1782) which can cause severe epithelial tissue damage at its predilection site at the ileocaecal junction and adjoining segments of the small and large intestine (Borgsteede and van Beek 1998; Kjaer et al. 2007; Rehbein et al. 2013; Nielsen 2016). In some regions, the non-pathogenic tapeworm *Anoplocephala magna* (Abilgaard, 1789) seems to be relatively abundant, whereas *Anoplocephaloides mamillana* (Mehlis, 1831) is reported only sporadically (Borgsteede and van Beek 1998; Rehbein et al. 2013).. Recognition of the potential clinical impact of *Anoplocephala* has compelled clinicians and researchers to employ more sensitive and precise diagnostics aimed at detecting infection to inform treatment applications that would mitigate the presence of pathogenic burdens.

Various diagnostic methods for the detection of equine tapeworms based on coprological, immunological or molecular data (Proudman and Edwards 1992; Höglund et al. 1995; Proudman and Trees 1996a, b a,b; Meana et al. 1998; Williamson et al. 1998; Gasser et al. 2005; Abbott and Barrett 2008; Skotarek et al. 2010; Rehbein et al. 2011; Tomczuk et al. 2014; Jürgenschellert et al. 2020; Traversa et al. 2008) are available. Traditionally, coprological methods were applied for the diagnosis of equine anoplocephalosis. However, tapeworm egg detection in horse feces is complicated by tapeworm egg-shedding patterns, with low numbers of eggs released sporadically in clumps within segments that are not evenly distributed throughout the fecal mass (Slocombe 1979; Gasser et al. 2005; Abbott and Barrett 2008; Nielsen 2016). For these reasons, the sensitivity of the commonly used coprological methods is low (Kjaer et al. 2007; Nilsson et al. 1995; Meana et al. 1998; Williamson et al. 1998). Proudman and Edwards (1992) reported 61% sensitivity of a centrifugation/flotation egg detection technique and a sensitivity of up to 92% for horses with more than 20 tapeworms, which they proposed distinguished horses suffering pathogenic (i.e., > 20 tapeworms) from those with non-pathogenic burdens; however these adaptations to the method would not be able to address the issue of intermittent egg shedding within proglottids. In addition, Rehbein et al. (2011) highlighted that a modified coprological ‘double centrifugation/combined sedimentation-flotation technique’ detected more tapeworm eggs when the volume of fecal sample was increased and concentrated sugar solution was used. The method showed a detection rate of up to 97.3% in fecal samples that had tested “positive” for *Anoplocephala* eggs using other coprological methods; however, its true sensitivity for patent infection remains unknown as only fecal egg detection was used as the comparator and no validation was undertaken using material from horses with enumerated tapeworm burdens. Despite the high specificity of coprology (98–100%), low egg shedding intensity, change of cestode generations, maturity/absence of gravid proglottids and other features of the parasite’s biology need to be taken into account (Lyons et al. 2018). It follows that tapeworm eggs in feces may be reliable markers to reveal potential parasite contamination onto pasture but, considering the disadvantages outlined above, coprological methods do not provide a reliable assessment of tapeworm infection levels in individuals and hence are not good predictors of disease risk (Trotz-Williams et al. 2008; Back et al. 2013; Lightbody et al. 2018).

The equine IgG(T) immunoglobulin subclass has been identified as a marker for infection for *A. perfoliata*, with a specific response to a 12/13 kDa excretory/secretory antigen used as the basis of a serum ELISA (Proudman and Trees 1996a, b; Proudman et al. 1998; Lightbody et al. 2016). In this ELISA, antigen specific IgG(T) levels correlate with the intensity of the tapeworm infection (Lightbody et al. 2016). From the clinical point of view, employment of antibody detection methods serves as an important tool in the diagnosis of equine anoplocephalosis for several reasons: early detection of infection, determination of a pathogenic infection and correlation with the degree of ileo-caecal or colon injury (Proudman et al. 1998). Lightbody et al. (2016) further developed and optimized the serum-based ELISA, increasing its sensitivity (85%), with slightly lower specificity (78%), and subsequently developed a saliva-based ELISA (EquiSal®) with high sensitivity (83%) and specificity (85%) that used the same antigen/antibody detection principle as the serum ELISA (Lightbody et al. 2016). A key advantage of the saliva-based ELISA is the relatively short half-life of antibodies in saliva resulting in a shortened persistence after effective treatment (Lightbody et al. 2016). Recently, the serum and saliva-based ELISAs were applied to study the prevalence of *Anoplocephala* spp. infection in horses in Germany (Jürgenschellert et al. 2020). These studies revealed a higher prevalence of tapeworm infection in horses detected by the serum- and saliva-based ELISA methods (prevalence 16.2% and 29.5%, respectively) compared to coprological techniques (0.6% prevalence). These results emphasize the advantage of using antibody-based methods for detecting tapeworm infection, especially in regions where current information on the prevalence of equine anoplocephaloses is scarce.

Depending on the method used, seasonal patterns of tapeworm occurrence have been described (Meana et al. 2005; Rehbein et al. 2013). Kjaer et al. (2007), Pittaway et al. (2014), Engell-Sørensen et al. (2018) and Sallé et al. (2020), reported highest infection rates in autumn/winter months. The latter is affected by the presence of intermediate host, soil oribatid mites (Denegri 1993). Regarding tapeworm occurrence in Slovak horses, published data from Slovakia and neighbouring countries (Szell et al. 1999; Königová et al. 2001; Gawor 2002; Kornaś et al. 2006, 2010; Vojtková et al. 2006; Pilaker and Goldová 2008; Tomczuk et al. 2014;) indicates a relatively low prevalence (0.8–25%) as detected by coprological methods; however, higher prevalence rates have been reported in Central Europe and other parts of the world (Gasser et al. 2005; Nielsen 2016; Jürgenschellert et al. 2020). This could be associated with a lack of reliable techniques to diagnose the presence of tapeworm infection (Gasser et al. 2005; Abbott and Barrett 2008; Lightbody et al. 2016; Nielsen 2016). Therefore, this study aimed to examine the occurrence of *Anoplocephala* infection on horses kept under different conditions by coprological and serum or saliva-based antibody detection methods. An analysis of various risk factors associated with the presence of infection in domestic horses in Slovakia was also undertaken.

## Material and methods

### Study design and horses included

**Fig. 1:**
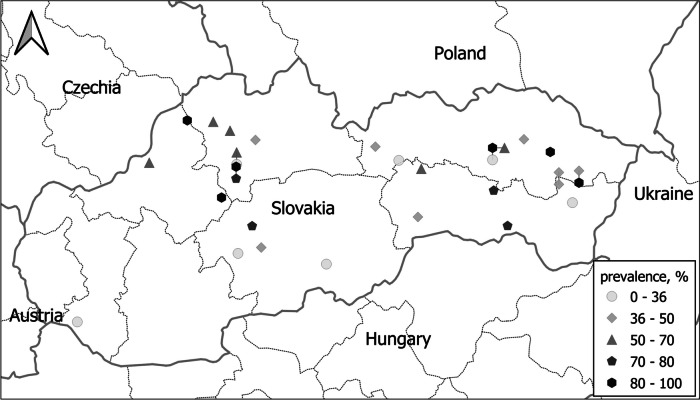
Distribution of examined farms in different regions of Slovakia; grade of the *Anoplocephala* spp. infection prevalence is indicated by colors

The study was carried out in September–December 2021 and in March–July 2022 in six regions of Slovakia (Fig. 1). A total of 427 horses of different age groups (3 months to 30 years-old) from 31 farms were included in the study (Table 1). Four age groups were categorized for the analysis: foals (up to 1 year-old), young horses (> 1–4 years-old), adult horses (> 4–15 years-old) and old horses (> 15 years-old). Of the 30 horse breeds included, 214 (50.11%) belonged to the Slovak Warmblood breed, while other breeds were represented by 1 to 20 individuals; therefore, the correlation between horse breed and levels of tapeworm infection was not analysed. The horses included were used mainly for recreational riding (45.19%), sport (30.21%), sport and breeding (18.26%) and agrotourism (2.34%). The cohort also included a group of horses kept in a shelter without any specific use (3.98%).

**Table 1:** Information on the 427 horses from 31 Slovakian farms included in the study

Parameter	Value		Total
	2021	2022	
Number of horses/farms	206 / 15	221 / 16	427 / 31
Age (range)	0.5–30 years	0.4–29 years	0.4–30 years
Foals (up to 1 year old) (%)	7 (3.39%)	6 (2.71%)	13 (3.04%)
Young horses (> 1–4 years old) (%)	32 (15.53%)	29 (13.12%)	61 (14.29%)
Adults (> 4–15 years old) (%)	133 (64.56%)	142 (64.25%)	275 (64.40%)
Old horses (> 15 years old) (%)	34 (16.50%)	44 (19.91%)	78 (18.27%)
Mares (%)	110 (53.39%)	93 (42.08%)	203 (47.54%)
Geldings (%)	84 (40.78%)	105 (47.51%)	189 (44.26%)
Stallions (%)	12 (5.83%)	19 (8.60%)*	31 (7.26%)*
Farm size:			
Small (< 10 horses)	39 (18.93%)	45 (20.36%)	84 (19.67%)
Medium (11–20 horses)	89 (42.20%)	64 (28.96%)	153 (35.83%)
Large (> 21 horses)	78 (37.86%)	112 (50.68%)	190 (44.50%)
Horses sampled per farm (range)	9–33	6–23	6–33
Size/number of horses per farm (range)	9–56	6–55	6–56
Pasture area/horse (ha) (range)	0.2–3.8 ha	0.5–5.0 ha	0.2–5.0 ha
Pasture access (%)			
– unlimited	90 (43.68%)	151 (68.33%)	241 (56.44%)
– limited	104 (50.48%)	70 (31.67%)	174 (40.75%)
– no access	12 (5.83%)	0	12 (2.81%)
Anthelmintic used for the last treatment:			
Ivermectin + Praziquantel (%)	115 (55.83%)	68 (30.77%)	183 (42.86%)
Ivermectin (%)	25 (12.14%)	38 (17.19%)	63 (14.75%)
Moxidectin + Praziquantel (%)	12 (5.83%)	54 (24.43%)	66 (15.46%)
Doramectin (%)	0	14 (6.33%)	14 (3.28%)
Fenbendazole (%)	8 (3.88%)	23 (10.41%)	31 (7.26%)
Levamizole/Levamizole + Oxyclozanide (%)	27 (13.11%)	0	27 (6.32%)
Pyrantel (%)	0	14 (6.33%)	14 (3.28%)
Unknown	19 (9.22%)	10 (4.52%)	29 (6.79%)
Annual treatment schedule (horses/farms):			
selective (%)	0	0	0
low number of treatments (< 1–2/year)	20/2	24/2	44/4
moderate number of treatments (2–3/year)	137/9	147/11	304/20
high number of treatments (> 3–4/year)	49/4	50/4	79/7
Period between last anti-tapeworm treatment and sampling (days; range)	289 (209–752)	180 (134–235)	237 (134–752)
Fecal samples (%)	198 (96.12%)	203 (91.86%)	401 (93.91%)
Saliva samples (%)	143 (69.42%)	156 (70.59%)	295 (69.08%)
Serum samples (%)	206 (100.00%)	218 (98.64%)	424 (99.30%)

^*^ – 4 horses in 2022 were of unknown sex

All information about horse age, breed, sex, size of farm, horse-keeping conditions and deworming programme was obtained from horse owners via a questionnaire on the day of sampling. According to this information, the following anthelmintics were used for the preceding treatment: ivermectin (IVM), praziquantel (PRZ) in combination with IVM (IVM + PRZ), moxidectin (MOX) in combination with PRZ (MOX + PRZ), doramectin, fenbendazole, levamisole or levamizole + oxyclozanide, pyrantel. Serum and fecal samples were collected from almost all horses (> 90%), with saliva samples obtained from 70% of the study population (Table 1).

### Coprological analyses

Fecal samples from all horses were obtained rectally or collected promptly after defecation, then labelled and delivered to the laboratory. Before examination, all samples were kept in a refrigerator (+ 4°C) for 1–2 days. Two coprological methods were performed to investigate *Anoplocephala* fecal egg counts (FEC); the mini-FLOTAC (Noel et al. 2017) and the double centrifugation/combined sedimentation-flotation technique (Rehbein et al. 2011). To carry out the mini-FLOTAC technique, 5 g of samples were placed into a fill-FLOTAC device and dispersed with a 45 ml volume of glucose-NaCl solution (s.g. 1.25). The mini-FLOTAC chambers were filled with the 1 ml suspension and the FEC was computed after 10 min of flotation as reported by Noel et al. (2017). The FEC was recalculated to obtain eggs per gram (EPG) numbers using a multiplication factor of five. To confirm the mini-FLOTAC-derived coprological results, a double centrifugation/combined sedimentation-flotation technique was conducted (Rehbein et al. 2011) with minor modifications. A total of 30 g of feces was homogenized in 60 ml of tap water and strained through a sieve of 250 μm in mesh size. Thereafter, two centrifugation processes followed and the tube with suspension filled with saturated sucrose solution (s.g. 1.28) and closed for 5 min. The screw caps of the tubes were flushed and the sediment was microscopically examined for the presence of *Anoplocephala* spp. eggs.

### Serum and saliva analyses

Serum samples were collected from 424 horses (Table 1) and analysed with the Horse Serum Tapeworm ELISA (Austin Davis Biologics Ltd, Northamptonshire, UK) (Lightbody et al. 2016). Serum tapeworm scores were calculated as low (< 2.70), borderline (2.70 to 6.30) and moderate/high levels (> 6.30). The borderline and moderate/high thresholds were considered positive, while the low threshold was considered negative.

Saliva samples were collected from 295 horses (Table 1) and examined using the EquiSal® Tapeworm Saliva Test (Austin Davis Biologics Ltd., Northamptonshire, UK) (Lightbody et al. 2016). EquiSal Tapeworm Saliva Test scores were calculated as low (< -0.09), borderline (-0.09 to 0.6) and moderate/high (> 0.60) levels. For the analysis, the borderline and moderate/high thresholds were considered positive, and the low threshold was considered negative.

### Data analyses

All data collected were initially organized using Microsoft Excel. Confidence intervals (95%) were calculated using the Stern or Wald method in the Quantitative Parasitology 3.0 software (Rózsa et al. 2000). McNemar's test was performed with the 'mcnemar.test()' function within the R software environment (R Core Team 2021).

### Risk factor analysis

The effects of variables associated with an individual horse were tested for the positive outcome of the saliva ELISA test on *Anoplocephala* spp. All data analyses were performed in the R environment (v 4.1.0) (R Core Team 2021) with R Tools 4.0 installed. The functions from the “tidyverse” package (Wickham et al. 2019) were used for data manipulation and visualization.

**Fig. 2:**
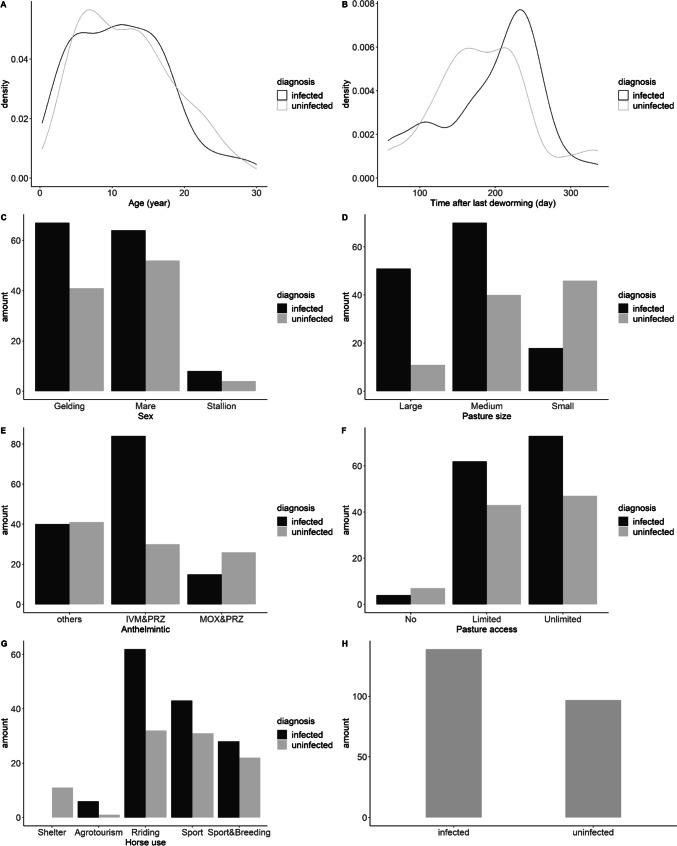
Ratio in positive and negative outcomes of the saliva ELISA tests for variables chosen as fixed effects for risk factor analysis model (A–G). The general ratio of negative and positive test cases; this variable is used as the model’s response (H). The numeric variables are visualized with density plots and categorical variables – bar plots. The total number of samples involved in this visualization is 236

Before modelling, only low-correlated, or uncorrelated variables were selected for further analysis. For this purpose, a heterogenous correlation matrix consisting of Pearson product-moment correlations between numeric variables, polyserial correlations between numeric and categorical variables, and polychoric correlations between categorical variables was computed with function *hetcor* from the “polycor” package (Fox 2022). Then, only pairs of variables with a correlation lower than 0.5 were chosen. The processed dataset included the following variables: age, sex, pasture size, anthelmintic, pasture access, horse use, and time after the last deworming. Next, the following modifications were executed to prepare the dataset for modelling: all rows with incomplete data were excluded, and statistical outliers in the “time after the last deworming” variable were adjusted to align with its median value. Also, as one of the primary research interests was to estimate the effect of PRZ on tapeworm infection in horses, all schemes of anthelmintic treatment that did not include PRZ were combined in the category “others.” Additionally, the relationship between each variable (horse age, sex, horse use mode, anthelmintics, time after the last deworming, pasture size, and access to pasture) and the ELISA test results were visualized in order to summarize the dataset descriptively (Fig. 2, A–G).

A binomial generalized mixed model (GLMM) was applied to assess the effect of predictors on the binary state variable (Fig. 1, H), describing if the ELISA tests detected infection with *Anoplocephala* or not. Accordingly, the model incorporated the following fixed effects: age (measured in years), sex (mares, geldings, stallions), pasture size (small, medium, large), anthelmintic (IVM + PRZ, MOX + PRZ, others), pasture access (no, limited, unlimited), horse use (recreational riding, sport, sport and breeding, agrotourism, shelter horse), time after the last deworming (measured in days). Additionally, the model incorporated farm identifiers as a random effect. The model was fitted within a Bayesian framework, using Markov Chain Monte Carlo (MCMC) as implemented in function *brm* from the “brms” package (Bürkner 2021). MCMC was run using four chains of 50000 iterations, each with 12500 iterations for each chain discarded as burn-in. For the model, normal priors with zero mean and standard deviation equal to five for all fixed effects were defined. These weak priors reflect the authors’ consensual belief based on their own experience of deworming and information from a recent paper (Jürgenschellert et al. 2020) that an effect size of a particular fixed effect should not exceed several dozen thousand times.

The correct classification rate and Area Under the Curve (AUC) were used to assess the model’s goodness of fit. Both parameters were computed based on the model’s posterior predictive probabilities using 0.5 as the threshold to interpret model responses as negative or positive. The AUC was estimated with function *roc* from the package pROC (Robin et al. 2011). For evaluating the proportion of the variance explained by the grouping structure, an intercept-only model was fitted with the farm identifiers as the mixed effect with a further calculation of an intraclass correlation coefficient (ICC) for this model with function ICC from the package “performance” (Lüdecke et al. 2021).

## Results

### Horse population

General data on the horse population is presented in Table 1. Most horses were adults (64.40%); only 3.04% of horses were less than 1 year-old. Mostly mares (47.54%) and geldings (44.26%) were included in the study. Available pasture sizes at the horse farms selected varied considerably, from 0.18 ha to more than 5.0 ha per horse. The horses were kept on large or medium-size farms with unlimited (56.44% of horses) or limited (40.75%) pasture access; only 12 horses from one horse farm (2.81%) did not have any access to pasture.

The horses selected were mostly sport and breeding horses (224 horses; 52.46%) and horses used for recreational riding (175 horses; 40.98%); one horse farm was a shelter for mistreated horses (16 horses; 3.74%) and on one farm, horses were kept for “agritourism” in mountain area (12 horses; 2.81%).

Macrocyclic lactones (IVM and MOX) were the most widespread anthelmintics used. Here, IVM, MOX and doramectin were used in 326 horses (76.34%) on 25 farms; while benzimidazoles (fenbendazole) or tetrahydropyridines (pyrantel) were used in only 31 horses (7.26%) on three farms and in 14 horses (3.28%) on one farm, respectively. PRZ, used for the treatment of tapeworm in combination with IVM or MOX, was administered to 249 horses (58.32%) on 19 farms.

From the questionnaire, it was clear that selective treatment, based on targeted therapy of diagnostically-proven infected horses, was not applied at any horse farm. Most horse owners employed strategic deworming of all horses 2–4 times a year without previous fecal examination. Irregular anthelmintic treatment less than 1–2 times per year was employed in 44 horses from 4 farms (10.30%); all of these horses were used for recreational riding or breeding. Most horses (71.19%) were dewormed regularly twice a year or, in some cases, three times per year, and 18.50% of horses, used for sport and leisure riding, were dewormed more than 3–4 times per year. Anti-cestode treatment was applied 1–2 times per year on 11 farms (73.33%) in 2021. In 2022, 62.50% owners reported PRZ use in their deworming protocol, whereas only one owner claimed random pyrantel treatment administration.

According to the information obtained, no regular fecal examination was performed at any horse farm. Coprological examination to assess anthelmintic efficacy was performed at three of the horse farms. However, most horse owners were aware of the development of anthelmintic resistance; 21 of 31 farms examined (67.74%) practised regular or periodical rotation of anthelmintics. The samples collected from six of eight regions of Slovakia (Fig. 1) revealed that *Anoplocephala* spp. infection is distributed throughout the whole country.

### Fecal examination

During the 2 years of study, 401 fecal samples were examined (Table 1). The mini-FLOTAC method, as well as the double centrifugation/combined sedimentation-flotation technique, confirmed corresponding results; the same horses were found to be positive for tapeworm infection with both methods. *Anoplocephala* spp. eggs were found in six samples in 2021 (prevalence = 3.01%, 95% CI 1.33–6.48%) and two in 2022 (prevalence = 0.99%, 95% CI 0.18–3.58%). Overall, the prevalence was 1.99% (95% CI 0.94–3.94%). At farm level, the prevalence of tapeworm eggs detected in feces was 12.90% (95% CI 4.54–28.82%). Horses found positive for *Anoplocephala* spp. eggs were geldings (7) or mares (1), mainly of the Slovak Warmblood breed (7 of 8 horses).

### Serum ELISA data

Of 424 serum samples examined with the serum-based tapeworm ELISA over 2 years, 167 samples (39.39%) tested positive (95% CI 34.78–44.21%) (Table 2). Among these, 61 horses were diagnosed as borderline (14.39%; 95% CI 11.30–18.13%) and 106 as moderate/high (25.0%; 95% CI 21.09–29.34%). At farm level, the prevalence was recorded as 83.87% (95% CI 66.37–93.42%), with at least one horse per farm reporting a borderline serum score with antibodies against *Anoplocephala* spp.

**Table 2:** Prevalence of *Anoplocephala* spp. in horses examined by different methods

Method	Number of positive / number of examined horses	Prevalence, %	95% confidence interval, %
Coprological*	8/401	1.99	0.94–3.94
Serum ELISA	167/424	39.39	34.78–44.21
Saliva ELISA	168/295	56.95	51.19–62.55

^*^Mini-FLOTAC and double centrifugation/combined sedimentation-flotation technique

The prevalence of *Anoplocephala* spp. in horses varied between the two years of sampling. Of 206 horses examined in September–December 2021, 91 (44.17%) tested positive (95% CI 37.36–51.21%), with 22 horses diagnosed as borderline (10.68%; 95% CI 6.96–15.71%) and 69 as moderate/high (33.50%; 95% CI 27.22–40.27%). Of 218 horses examined in March–July 2022, 76 (34.86%) tested positive (95% CI 28.64–41.50%), with 39 horses diagnosed as borderline (17.89%; 95% CI 13.25–23.58%) and 37 as moderate/high (16.97%; 95% CI 12.41–22.66%).

### Saliva ELISA data

Of 295 saliva samples of horses from 31 farms examined with the saliva-based tapeworm ELISA over 2 years, 168 samples (56.95%) tested positive (95% CI 51.19–62.55%) (Table 2). Among them, 25 horses were diagnosed as borderline (8.47%; 95% CI 5.71–12.32%) and 143 as moderate/high (48.47%; 95% CI 42.71–54.24%). At farm level, 96.77% (95% CI 82.85–99.83%) were positive, with at least one horse per farm reporting a borderline serum score with antibodies against *Anoplocephala* spp.

As in the case of the serum-based tapeworm ELISA test, tapeworm prevalence varied between the two years of sampling; of 143 horses examined in September–December 2021, 89 (62.24%) tested positive (95% CI 53.86–69.97%), with seven horses diagnosed as borderline (4.9%; 95% CI 2.32–9.7%), and 82 as moderate/high (57.34%; 95% CI 48.95–65.42%). Of 152 horses examined in March–July 2022, 79 (51.97%) tested positive (95% CI 44.0–59.88%), with 18 horses diagnosed as borderline (11.84%; 95% CI 7.47–18.02%) and 61 as moderate/high (40.13%; 95% CI 32.53–48.35%).

### Comparison of coprological, serum, and saliva testing

During the two years of the study, the results of 295 matching samples from the fecal analysis and serum- and saliva-based tapeworm ELISA’s were obtained. Each horse found to be positive for *Anoplocephala* spp. according to fecal examination, had a moderate/high score in the saliva-based ELISA and a moderate/high score (6 horses) or borderline (1 horse) score in the serum-based ELISA.

**Fig. 3:**
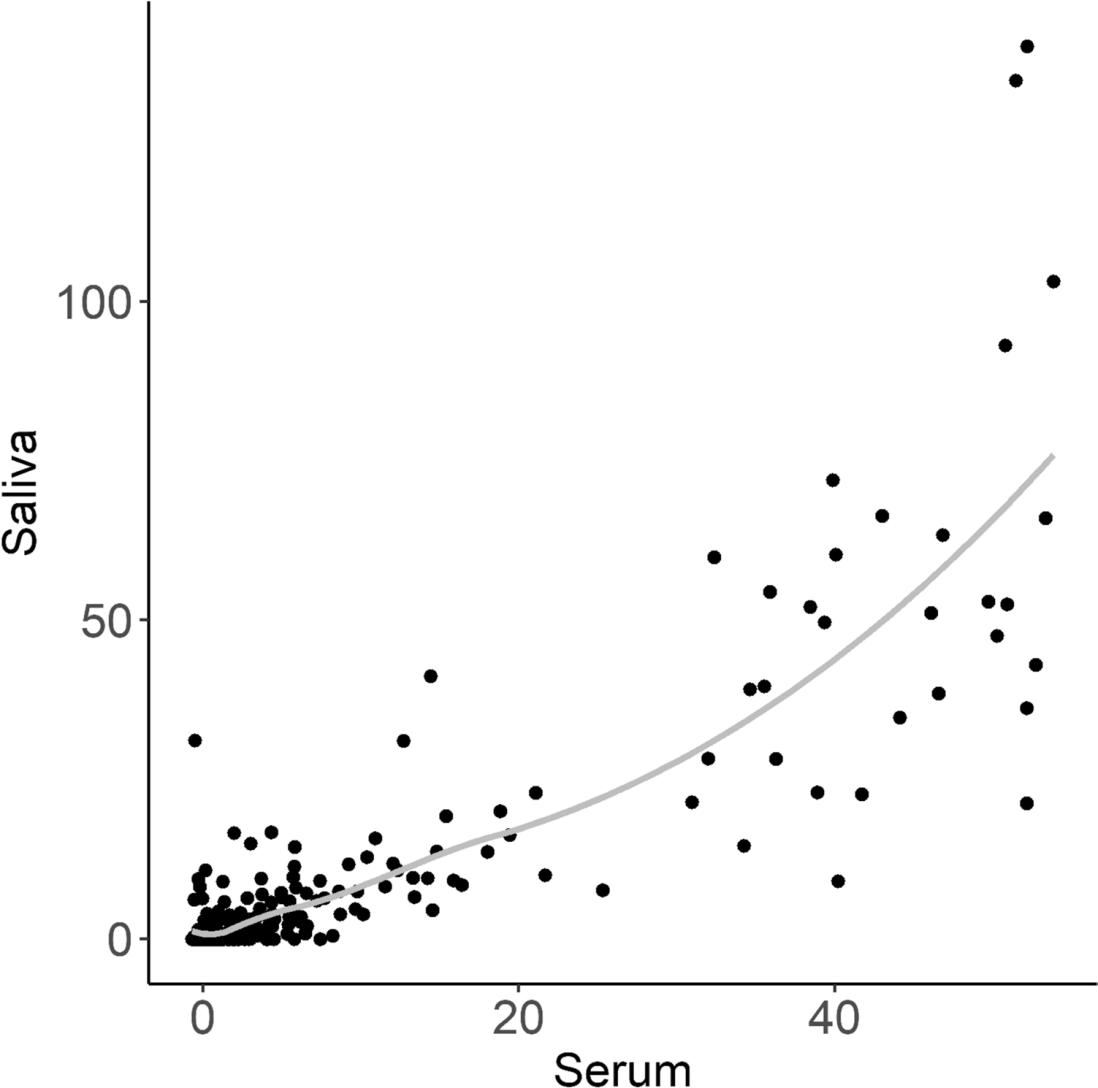
Relationship between results of the serum and saliva ELISA tests. Each dot represents one tested horse. The light-grey curve shows the general population trend built using the loess regression algorithm. The visualization includes 295 samples

Serum and saliva-based ELISA scores were plotted against each other (Fig. 3). Among the 295 matching samples, 168 (56.95%, 95% CI 51.19–62.55%) were positive in the saliva-based ELISA, and 108 (36.61%, 95% CI 31.17–42.36%) were positive in the serum-based ELISA. There was a highly significant positive Spearman’s rank correlation between serum and saliva scores (*ρ* = 0.75, *p* < 0.001) (Fig. 3). However, in the classification of cases (low versus borderline and moderate/high) between the serum and saliva-based ELISAs, there is a significant difference (McNemar’s test; *p*-value <  < 0.001). The serum-based ELISA diagnosed more samples as low compared to the saliva-based ELISA. General data on the prevalence of *Anoplocephala* spp. on the farms examined based on the serum- and saliva-based ELISA results are presented in Table 3.

**Table 3:** Prevalence of *Anoplocephala* spp. on 31 farms examined based on the serum- and saliva-based ELISA

Farm	Total number of horses	Serum-based ELISA			Saliva-based ELISA		
		N	Prevalence, (%)	95% CI, (%)	N	Prevalence, (%)	95% CI, (%)
#1	10	10	50.00	22.25–77.75	10	70.00	38.06–91.2
#2	40	15	6.67	0.35–30.20	10	0.00	0.00–29.08
#3	16	14	71.42	42.57–89.59	10	100.00	70.92–100.00
#4	9	9	55.55	25.14–83.12	5	80.00	34.26–98.97
#5	20	18	5.55	0.29–27.13	10	40.00	15.01–70.91
#6	12	11	27.27	7.99–59.55	10	70.00	38.06–91.27
#7	13	10	0.00	0.00–29.08	10	50.00	22.25–77.75
#8*	9	9	77.78	44.18–95.89	9	100.00	67.67–100.00
#9	40	12	16.67	3.05–45.71	11	36.36	13.51–66.71
#10	14	14	28.57	10.41–57.43	10	20.00	3.68–55.35
#11	18	17	94.12	71.27–99.69	18	100.00	81.48–100.00
#12*	5	5	100.00	50.00–100.00	5	100.00	50.00–100.00
#13	56	33	96.97	83.89–99.84	18	100.00	81.48–100.0
#14	12	12	33.33	12.29–62.98	7	85.71	44.58–99.26
#15*	11	11	36.36	13.51–66.71	8	50.00	19.30–80.70
#16	10	10	30.00	8.73–61.94	10	50.00	22.25–77.75
#17	9	9	33.33	9.78–67.66	9	44.44	16.88–74.86
#18	21	20	60.00	37.23–79.10	10	70.00	38.06–91.27
#19*	55	20	60.00	37.23–79.10	10	80.00	44.65–96.32
#20	35	14	28.57	10.41–57.43	10	80.00	44.65–96.32
#21	22	22	18.18	6.46–38.89	15	73.33	46.58–90.33
#22	14	13	76.92	48.04–93.39	7	85.71	44.58–99.26
#23	6	6	0.00	0.00–41.10	6	33.33	6.29–72.86
#24	14	9	0.00	0.00–32.30	9	11.11	0.57–44.34
#25	25	14	50.00	23.82–76.18	10	50.00	22.25–77.75
#26	9	9	55.55	25.14–83.12	7	71.43	34.13–94.66
#27	15	9	11.11	0.57–44.34	9	22.22	04.11–55.82
#28	16	12	0.00	0.0–24.30	12	0.00	0.00–24.30
#29	50	24	37.50	20.38–58.48	8	62.50	28.93–88.88
#30	18	16	12.50	2.27–37.16	10	60.00	29.09–84.99
#31	11	10	40.00	15.01–70.91	10	40.00	15.01–70.91

N – number of horses examined; * – farm with horses positive for *Anoplocephala* spp. in the coprological analysis are in bold

### Risk factor analysis

The evaluation of the intercept-only GLMM, with season and farm id used as random effects, showed a moderate ICC of 0.49, indicating that about 49% of the variability in the data can be attributed to the differences across seasons and farms.

**Fig. 4:**
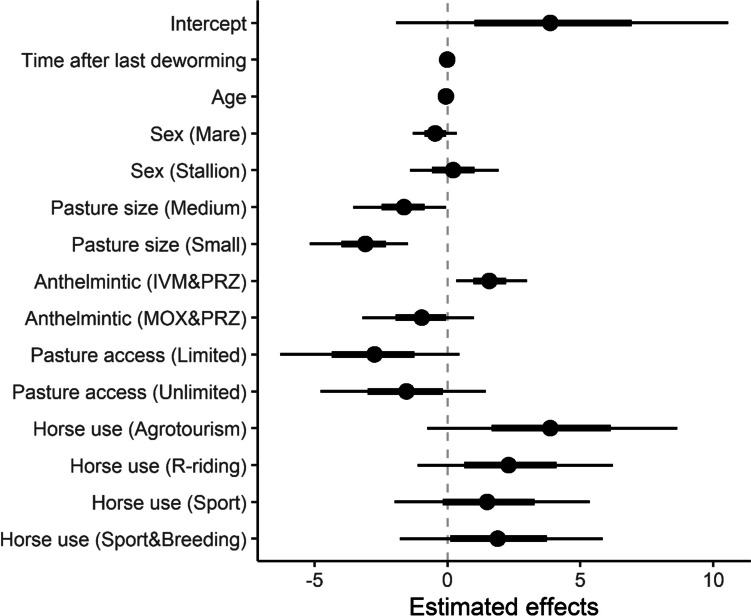
The summary of estimated posterior distributions for fixed effects in the binary logistic regression model used to explain positive samples (ELISA) of *Anoplocephala* spp. in serum. Circles show posterior mean, thick bars show standard error ranges of the posteriors, and thin lines show 95% credible intervals. A positive effect value indicates an increased probability of revealing *Anoplocephala* spp. infection and a negative effect value decrease this probability. An overlapping of credible intervals with zero assumes effects as unmeaningful. This analysis was based on 236 samples

The GLMM analysis identified several explanatory variables that meaningfully influenced the likelihood of obtaining positive test results (Fig. 4 and Table 4). The age of the horses meaningfully decreased the likelihood of receiving positive test results: older horses were less likely to yield positive test results. Pasture size also appeared to play a role; horses kept on medium and small-sized pastures were less likely to test positive than those on larger pastures. Regarding effect of the most recent anthelmintic treatment, the combination of IVM + PRZ increased the likelihood of positive results compared to the treatment schemes that did not include PRZ. In contrast, the treatment scheme of MOX + PRZ had no significant impact on test outcome compared to the treatments that did not contain PRZ. Access to pasture was found to be another meaningful factor. Horses with limited pasture access were less likely to yield positive test results compared to those with no access to pasture. In contrast, cases with unlimited pasture access showed no meaningful differences in test outcomes compared to the fully restricted group. It is necessary to point out that the upper edge of the credible interval for the age covariate was very close to zero; therefore, the interpretation of the factor as meaningful should be considered with caution as there is a chance that the real impact of the factors would be very close to the random.

**Table 4:** Fixed effects of the binary logistic regression model used to explain positive samples (ELISA) of *Anoplocephala* spp. in saliva. This analysis was based on 236 samples. Names of covariate levels with credible intervals that do not overlap with zero and, therefore, can be considered meaningful predictors are highlighted in bold

Covariate	N	Estimate	Odds ratio	95% credible interval
Time after the last deworming	236	-0.01	0.99	-0.01 – 0.00
Age	236	-0.06	0.94	-0.12 – 0.00
Sex				
Gelding	108	(Ref.)	(Ref.)	(Ref.)
Mare	116	-0.54	0.58	-1.37 – 0.28
Stallion	12	0.17	1.19	-1.48 – 1.87
Pasture size				
Large	62	(Ref.)	(Ref.)	(Ref.)
Medium	110	-2.17	0.11	-3.88 – -0.57
**Small**	**64**	**-3.49**	**0.03**	**-5.34 – -1.82**
Anthelmintic				
Others	81	(Ref.)	(Ref.)	(Ref.)
**IVM & PRZ**	**114**	**1.40**	**4.06**	**0.34 – 2.52**
MOX & PRZ	41	-1.25	0.29	-3.13 – 0.55
Pasture access				
No access	11	(Ref.)	(Ref.)	(Ref.)
**Limited**	**105**	**-3.79**	**0.02**	**-7.04 – -0.55**
Unlimited	120	-3.12	0.04	-6.36 – 0.11
Horse use				
Shelter	11	(Ref.)	(Ref.)	(Ref.)
Agrotourism	7	2.65	14.15	-1.91 – 7.35
R-riding	94	2.84	17.12	-0.51– 6.71
Sport	74	1.57	4.81	-1.76 – 5.40
Sport & Breeding	50	1.95	7.03	-1.64 – 5.87

The correct classification rate of the model was 79%; meanwhile, the AUC was equal to 0.76. Such values of both parameters indicate that discrimination of the model was acceptable.

## Discussion

This study presents new data on the distribution of *Anoplocephala* spp. on horse farms in Slovakia. Previous studies on the occurrence of equine parasites, including tapeworms, were carried out locally (Königová et al. 2001; Pilaker and Goldová 2008) and did not provide comprehensive information on the distribution of these parasites in this country. Horses from six of eight regions of Slovakia were included here; therefore, the results allow reliable conclusions to be made on the wider distribution of *Anoplocephala* spp. on horse farms throughout Slovakia.

Previously published data from Slovakia, the Czech Republic and Poland indicated a lower prevalence of *Anoplocephala* spp. on horse farms (Szell et al. 1999; Königová et al. 2001; Gawor 2002; Kornaś et al. 2006, 2010; Vojtková et al. 2006; Pilaker and Goldová 2008; Tomczuk et al. 2014). In these studies, various coprological methods were employed for the diagnosis of equine anoplocephalosis. Most researchers have noted the relative low sensitivity of these methods due to the fact that tapeworm eggs tend to be released at irregular intervals in clumps within segments and exhibit an uneven distribution in feces (Slocombe 1979; Gasser et al. 2005; Abbott and Barrett 2008; Nielsen 2016). According to the results of coprological examinations, only 0.1–2.8% of examined horses have been found to be infected by *Anoplocephala* spp. in Slovakia (Königová et al. 2001; Pilaker and Goldová 2008), 2.1–7.4% in Poland (Gawor 2002; Kornaś et al. 2006, 2010; Tomczuk et al. 2014), 6.7% in Czech Republic (Vojtková et al. 2006), ~ 0.6% in Hungary (Szell et al. 1999), and 3.0–6.3% in Germany (Epe et al. 2001; Hinney et al. 2011; Jürgenschellert et al. 2020). These figures are likely not to reflect the actual prevalence of anoplocephalosis as there are substantial differences in prevalence levels when coprological-derived results are compared to data derived post-mortem where substantially higher infection rates were identified when *A. perfoliata* larval and adult parasites were enumerated (Lyons et al. 2018; Gasser et al. 2005; Abbott and Barrett 2008; Rehbein et al. 2013; Tomczuk et al. 2015; Nielsen 2016). The results of the present study also support low sensitivity of the coprological techniques in comparison with the results of the serum and saliva ELISA tests. Only 1.99% of horses from 12.90% of the examined farms were found to be infected according to the coprological methods, while the results of the serum and saliva ELISAs revealed 39.39% and 56.95% horses positive for *Anoplocephala* spp., respectively. Two coprological methods, the mini-FLOTAC and the double centrifugation/combined sedimentation-flotation technique (Noel et al. 2017; Rehbein et al. 2011) showed similar results in complete agreement with recent German data (Jürgenschellert et al. 2020). Despite the information that the double centrifugation/combined sedimentation-flotation technique is relatively more sensitive compared to other flotation methods (Rehbein et al. 2011), it was difficult to draw a reliable conclusion here due to the low number of positive horses detected by the coprological methods.

The results here support the theory that due to low sensitivity of traditional coprological methods, the prevalence of *Anoplocephala* spp. infection based on fecal egg count (FEC) data often may be underestimated; therefore, the real tapeworm burden on horse farms assessed by these techniques is uncertain. Accordingly, a lack of reliable data and, consequently, insufficient attention to tapeworm control has resulted in an increase in problems associated with equine anoplocephaloses in Europe and around the world (Matthews et al. 2004; Gasser et al. 2005; Rehbein et al. 2013; Tomczuk et al. 2014; Nielsen 2016; Jürgenschellert et al. 2020). Antibody-based tests have been developed to provide better evidence of tapeworm occurrence (Lightbody et al. 2016, 2018; Jürgenschellert et al. 2020). Initial validation of the serum and saliva ELISAs used here (Lightbody et al. 2016) demonstrated strong positive correlations (Spearman’s rank correlation *ρ* = 0.78) of serologically detected antibodies to tapeworm burden from necropsy data. Saliva ELISA scores showed a similar pattern (Spearman’s rank correlation *ρ* = 0.74), as well as a strong positive relationship with serum ELISA results (Spearman’s rank correlation *ρ* = 0.86) (Lightbody et al. 2016). The results of serum and saliva ELISA tests obtained in the present study showed a relatively high prevalence of *Anoplocephala* spp. infection on horse farms (39.39% and 56.95% positive horses, respectively). In comparison to previously published research performed in Germany, the occurrence of this parasite in Slovak horses was higher (Jürgenschellert et al. 2020) but the data here is comparable to prevalence rates derived from post-mortem analysis of tapeworm burdens in other studies in Europe (Fogarty et al. 1994; Nilsson et al. 1995; Morgan et al. 2005; Kjaer et al. 2007; Rehbein et al. 2013; Pittaway et al. 2014; Tomczuk et al. 2015; Lightbody et al. 2016) and elsewhere (Sangioni et al. 2000; Chapman et al. 2002; Nielsen 2016; Gasser et al. 2005). Moreover, use of serum and saliva ELISAs on the same farms showed that application of the saliva assay revealed a higher number of positive horses (Table 2) and a higher prevalence of *Anoplocephala* infection at farm level (Table 3). This could be due to the saliva ELISA detecting more horses with low intensity infections (i.e., < 20 tapeworms per horse) compared to the serum-based assay as previously indicated (Lightbody et al. 2016). Regardless, direct comparison of the results of the serum and saliva ELISA tests revealed a highly significant positive correlation (Spearman’s rank correlation *ρ* = 0.75, *p* < 0.001), in line with previous results from the UK (Lightbody et al. 2016) and Germany (Jürgenschellert et al. 2020). It should be considered that as these are antibody-based tests with 78–85% specificity, false positive results may arise in some cases due to the presence of parasite-specific antibodies stimulated by previous tapeworm infection prior to treatment. For this reason, it is recommended not to use these tests within 12 weeks (saliva test) and 4 months (serum test) of an anti-cestode anthelmintic treatment.

From the clinical viewpoint, employment of antibody detection methods serves as an important tool in the diagnosis of equine anoplocephalosis for several reasons: early detection of infection, supporting the determination of a potentially pathogenic burden (> 20 tapeworms) and correlation with the degree of ileo-caecal or colon injury (Proudman et al. 1998). Nevertheless, a disadvantage of this ELISA is cross-reactivity of *A. perfoliata* with *A. magna* antigens; thus, it cannot be certainly presumed which particular species is harbored (Bohórquez et al. 2012). Despite this, both ELISA analyses remain beneficial diagnostic techniques that inform tapeworm control by sensitively detecting infected individuals (Bohórquez et al. 2012; Lightbody et al. 2016, 2018; McGhee and Fujihashi 2012).

Visualization of the initial data on tapeworm infection of horses of different ages, and sexes and kept under various management/use systems showed interesting tendencies (Fig. 4). The ratio of infected/uninfected stallions and mares was lower than the ratio of infected/uninfected geldings. Horses used for recreational riding had the highest infected/uninfected ratio compared to horses used for other activities. Moreover, analysis of the effect of various anthelmintics on *Anoplocephala* spp. infection showed unexpected results—the highest infected/uninfected ratio was observed on farms where horses had been administered with IVM + PRZ at their preceding anthelmintic treatment. Next, binomial GLMM analysis was undertaken to assess the influence of various factors on infection with *Anoplocephala* spp. Factors such as horse sex and horse use did not have a significant effect on the level of infection. It could be that, in Slovakia, horse-keeping conditions and management for horses of different age/sex groups, as well as forms of use, do not differ as much as in other countries (Kornaś et al. 2010; Fritzen et al. 2010; Kuzmina et al. 2016; Nielsen et al. 2016, 2018; Hedberg-Alm et al. 2020). According to the GLMM analysis, four factors had a significant influence on infection: horse age, pasture size, access to pasture, and anthelmintic treatment scheme. The negative effect of small pasture size on tapeworm infection can be explained by the fact that populations of oribatid mites, the intermediate hosts of *Anoplocephala* spp., have been found to be at lower levels on smaller over-grazed pastures compared to larger ones (Hubert 2000; Chachaj and Seniczak 2005; Corral-Hernández and Iturrondobeitia 2012). Accordingly, it can be proposed, here, that a reduced oribatid mite population could lead to a decrease in tapeworm transmission in horses grazed on smaller paddocks. The influence of factors such as access to pasture on infection that appeared to be significant is similar to the results of a study in Germany (Jürgenschellert et al. 2020). Apparently, in this case, even a limited grazing time on contaminated pasture was sufficient for horse infection with *Anoplocephala* spp.

A positive relationship of the application of the IVM + PRZ combination with tapeworm infection in Slovak horses here is in contrast to what has been identified in a recent German study (Jürgenschellert et al. 2020). Since PRZ is considered to be very effective against tapeworms (Lyons et al. 1995; Rehbein et al. 2003; Barrett et al. 2004; Gasser et al. 2005; Slocombe et al. 2007; Nielsen 2016), it is recommended for treating *A. perfoliata* worldwide. According to the observations here, the combination of IVM + PRZ is the most widely used anthelmintic in Slovak horse farms. Nevertheless, even a preliminary assessment of the ratio of infected/uninfected horses (Fig. 1) showed that the highest number of infected horses was observed on farms where IVM + PRZ were used. There may be several reasons for this phenomenon, including underdosing of anthelmintic. Importantly, the combination of IVM + PRZ was usually applied on farms where anoplocephalosis had been previously diagnosed and identified as prevalent. The PRZ chemical has no persistent effect against tapeworm, and whilst cysticercoid stages in oribatid mites remain on pasture, reinfection of horses grazing contaminated paddocks is rapid. Therefore, the present observation of a higher level of infection may not be a consequence of the use of IVM + PRZ anthelmintic per se but could reflect a higher level of tapeworm transmission on these farms. In Slovakia, most horse owners usually carry out deworming of their horses with no assistance from qualified veterinarians. Thus, the probability of incorrect calculation of drug dosage is relatively high, which will affect the efficacy of the anthelmintic. In addition, the results obtained may suggest a possibility of anthelminthic resistance development in *Anoplocephala* spp. in Slovakia due to regular underdosing (Kaplan 2004; Matthews et al. 2004; Nielsen et al. 2022). Further studies will be required to prove this assumption and, in this regard, it is notable that possible resistance in *A. perfoliata* to PRZ and pyrantel pamoate has recently been reported in the US (Nielsen 2023).

In recent decades, equine tapeworm infection reports in horses in Europe has increased considerably (Kornaś et al. 2010; Rehbein et al. 2013; Tomczuk et al. 2014, 2015; Jürgenschellert et al. 2020), possibly due to intensive use of benzimidazoles and macrocyclic lactone drugs, which are not effective against tapeworms (Nielsen et al. 2013). Therefore, the relatively recent widespread application of PRZ on horse farms in Slovakia could not essentially have yet reduced levels of *Anoplocephala* infection in horses. More evidenced-based use of PRZ on horse farms in Slovakia for a longer period, supported by diagnostic monitoring of infection using appropriately sensitive methods, will likely reduce equine tapeworm infection in this country.

## Data Availability

The original datasets are available upon request to the corresponding author.
